# Six-implant-supported immediate fixed rehabilitation of atrophic edentulous maxillae with tilted distal implants

**DOI:** 10.1186/s40729-017-0096-0

**Published:** 2017-07-25

**Authors:** S. Wentaschek, S. Hartmann, C. Walter, W. Wagner

**Affiliations:** 1 Department of Prosthetic Dentistry, University Medical Center of the Johannes Gutenberg-University Mainz, Augustusplatz 2, 55131 Mainz, Germany; 2 Department of Oral and Maxillofacial Surgery - Plastic Surgery, University Medical Center of the Johannes Gutenberg-University Mainz, Augustusplatz 2, 55131 Mainz, Germany

**Keywords:** Tilted implants, Edentulous maxilla, Full-arch prostheses, Immediate loading, Implant stability quotient, Periotest

## Abstract

**Background:**

The aim of this retrospective study was to evaluate the treatment outcome of six Bredent blueSky™ implants (Bredent GmbH, Senden, Germany) immediately loaded with a fixed full-arch prosthesis (two tilted posterior and four axial frontal and premolar implants).

**Methods:**

All 10 patients with atrophic edentulous maxillae being treated with a standardized procedure from 09/2009 to 01/2013, who had a follow-up of at least 3 years, were included. Sixty implants were placed to support 10 screwed prostheses. Twenty-one of them were inserted in fresh extraction sockets. Lab-side-prepared provisional fixed prostheses were placed at the day of implantation. Periotest (PT) values and implant stability quotient (ISQ) were measured after implant surgery and after 3 months of healing in all patients.

**Results:**

The analyzed implants were in function in mean 64 ± 13 months (range 42 to 84 months). One axial and two tilted implants failed in three patients. The mean PT values decreased, and ISQ increased significantly after the first 3 months at the osseointegrated tilted and axial implants. With an area under the curve of 0.503 and 0.506 in the receiver operating characteristic, the PT values and the ISQ were unspecific parameters and unsuitable as a predictor for the risk of non-osseointegration.

**Conclusions:**

Within the limits of this small group (*n* = 10 patients/60 implants), the failure rate of the analyzed implant system (*n* = 3 respective 5% implant loss) seems to be comparable with other immediate-loading protocols. The failure rate of tilted implants in the atrophic upper jaw was quite high, but the aimed treatment concept could be achieved in every patient. The rehabilitation of the posterior region in edentulous maxilla remains a challenge.

## Background

For a few years, there has been a trend towards minimally invasive implant treatment concepts avoiding bone augmentation even in very atrophic edentulous jaws. These concepts aim to make an implant treatment with a shorter duration, with less inconvenience such as swelling or pain and possibly also economically more attractive [1]. If the implant treatment is less invasive, because of the possible smaller surgical risks and lower costs, implant therapy can be provided for a larger number of patients. Minimally invasive mainly means the adaptation of the implant dimension or position to the existing anatomy to avoid bone augmentation procedures [1]. One possible strategy to avoid augmentations in the distal atrophic maxilla is to insert short implants. In recent reviews, implants of less than 10 mm are not inferior to longer implants relating to bone loss or survival rate [2–4]. But also for the insertion of short implants, the bone height in the atrophic posterior maxilla is often not enough [5].

An alternative to short implants are longer tilted implants [6] with a possibly higher primary stability combined with the posterior position of the implant shoulder [7–9]. These characteristics seem to make them especially suitable for immediate loading in the edentulous jaws [10] as it is often performed [5]. This treatment concept with loading on the same day appears to achieve high patient satisfaction [1], but there are also some disadvantages. Tilted implants might be more difficult to insert and need technical angulated abutments. To position the implants in an optimal position parallel to the anterior sinus wall, a computer-guided implant planning and navigated insertion is more often needed.

Different implant systems have been investigated using the concept of tilted implants [11], but due to the different geometric properties and prosthetic components, they may behave differently, so that all systems used for this concept must prove their suitability. Because this implant type was previously rarely investigated in the concept of immediate loading [12], the aim of this retrospective study is to evaluate the success rate of Bredent blueSky™ implants (Bredent GmbH, Senden, Germany) in immediate full-arch loading with tilted posterior implants using minimal invasive surgery. In addition to the osseointegration and bone loss, the stability parameters’ implant stability quotient (ISQ; measured by resonance frequency analysis (RFA)) and Periotest (PT) values were compared between tilted and axial implants and their changes after osseointegration were recorded. The suitability of the chosen combination of implants, abutments, and materials for the provisional restorations after use in a clinical setting should be examined.

## Methods

### Patients

In a retrospective study, all patients with immediately loaded implants in an edentulous maxillae with limited posterior ridge dimensions that received an equal concept were included if they had a follow-up of at least 3 years. The concept contained immediate loading with distal tilted implants and six implants per edentulous maxillae of a single implant system (blueSky™ implants, Bredent GmbH, Senden, Germany), and it includes an equal lab-side-prepared provisional fixed prosthesis.

All patients have received implant stability parameter measurements that were routinely collected at immediate loading directly after implant insertion and after first removal of the provisional restoration 3 months after surgery. The ISQ after RFA and PT values were measured.

The retrospective data analysis was conducted in accordance with the Helsinki Declaration of 1975, as revised in 2008, and all patients signed an informed consent. After consulting the local ethic committee, the decision was that due to the retrospective character of this study with no additional data acquisition, no ethical approval is needed according to the hospital laws of the appropriate state (Landeskrankenhausgesetz Rhineland Palatinate, Germany).

### Selection criteria

Patients who were treated with this concept had to have the desire and the indication for an implant-supported full-arch prosthesis and concerns regarding bone-grafting procedures. They had to be physically and psychologically capable of undergoing conventional implant surgery. They had to have a reduced bone volume in the molar region of the maxilla that would not allow placing dental implants of at least 6 mm in length without bone augmentation. But placement of tilted implants in the area of the premolars with an implant length of at least 10 mm had to be possible so that the implant was surrounded by bone. All patients had to be treated by the same maxillofacial surgeon and the same prosthodontist.

The exclusion criteria were an active infection or inflammation at the intended implants sites; major systemic disease, e.g., uncontrolled diabetes mellitus, radiation, or chemotherapy within 5 years prior to the surgery; bone-physiology-changing drugs such as bisphosphonates, severe bruxism, or clenching habit; and poor oral hygiene.

### Presurgical phase

The patients were screened with preliminary panoramic radiographs, and since all the implants were 3D planned (SKYplanX™ program, Bredent GmbH, Senden, Germany) and inserted with a guiding template, a cone-beam CT (CBCT) was obtained eventually (KaVo 3D eXam™ unit, KaVo Dental GmbH, Biberach/Riss, Germany).

### Surgical procedure

**Fig. 1:**
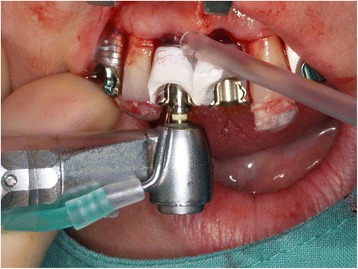
Preparation of implant cavity through corresponding metal sleeves after extraction of the central incisors using a surgical template supported by hopeless remaining teeth

The drillings were performed using a 3D-planned surgical template with different metal sleeves corresponding to the diameter of the drills (Fig. 1). Implants were inserted torque controlled under vision without the surgical template. Primary implant stability was assessed immediately following implant insertion by PT (Medizintechnik Gulden, Modautal, Germany) and RFA (Osstell, Gothenburg, Sweden).

### Prosthetic procedure

#### Immediate implant loading

Definitive titanium abutments (0°, 17.5°, 35°; fast & fixed abutments, Bredent, Senden, Germany) were attached to the implants. The abutment screws were tightened with a torque of 25 N cm. On these abutments, impression copings for closed trays were seated and an impression and a provisional inter-jaw relationship recording with a silicone were performed.

**Fig. 2:**
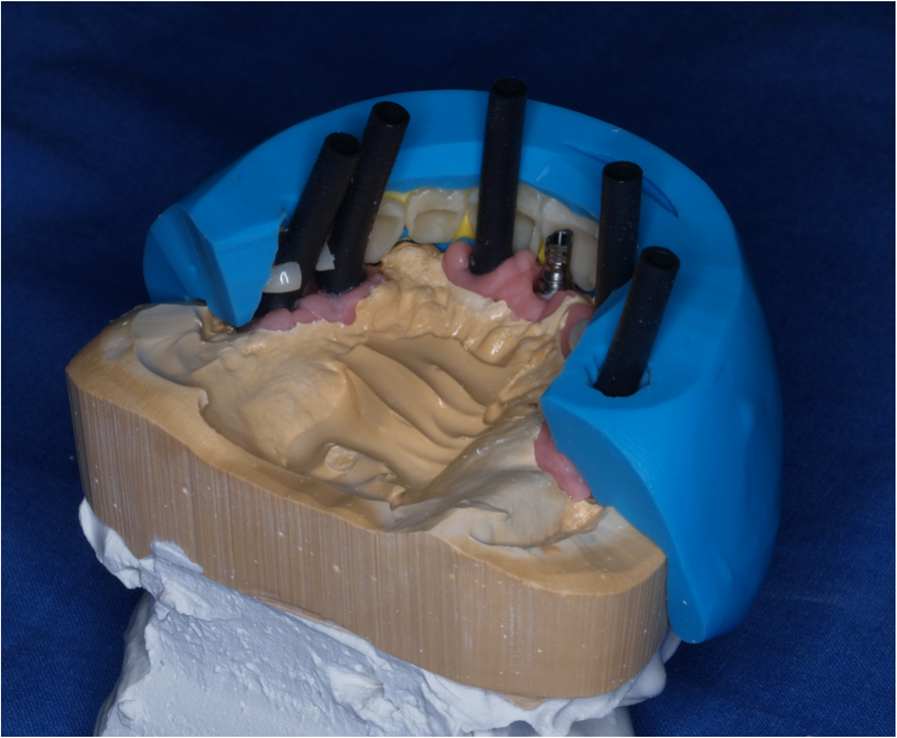
Preparation of the composite veneers for making the temporary restoration

**Fig. 3:**
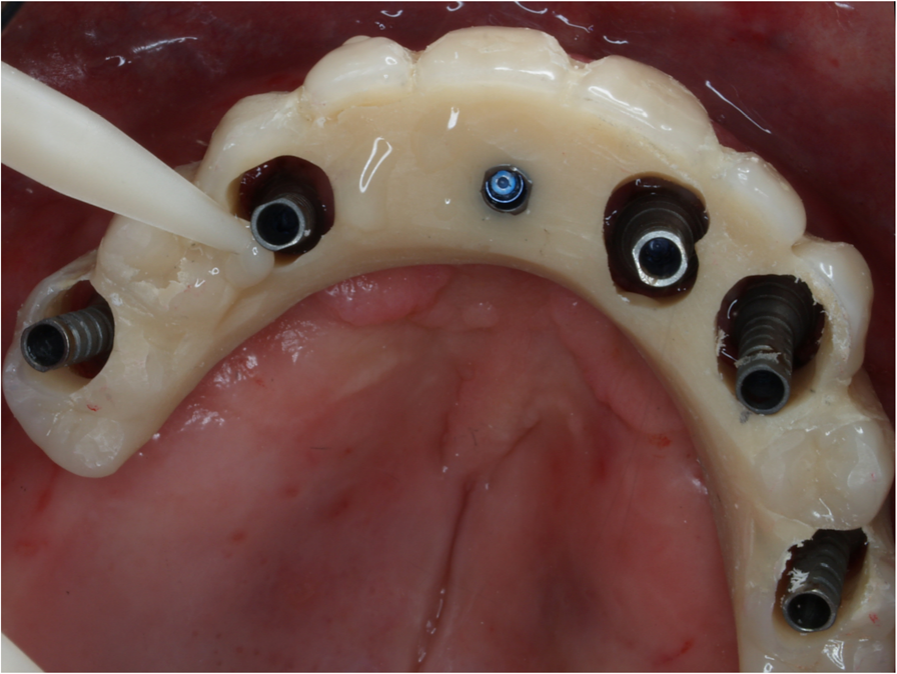
Fill-in of the occlusal perforations with self-curing resin to connect the prostheses to the temporary titanium cylinders

After cast making, temporary resin prostheses using a composite veneering system (visio.lign, Bredent, Senden, Germany) were prepared in the laboratory (Fig. 2). These temporary restorations were perforated in five of the six implant regions. After the temporary prosthetic titanium cylinders (Bredent, Senden, Germany) were attached on the abutments and the resin superstructures were placed over the cylinders, the superstructure perforations were filled with self-curing resin (Qu-resin™; Bredent, Senden, Germany) (Fig. 3). The superstructure was removed, completed, and relined. The provisional restoration was inserted, the screw holes were sealed, and the denture was adjusted on the occlusal plane. All provisional prostheses were inserted on the same day of implant insertion. With the provisional restorations, no further distal tooth was replaced than that under which the distal implant was positioned. Therefore, the distal cantilever extensions of the provisional prosthesis have not exceeded the width of a half molar.

### Post-surgical phase

**Fig. 4:**
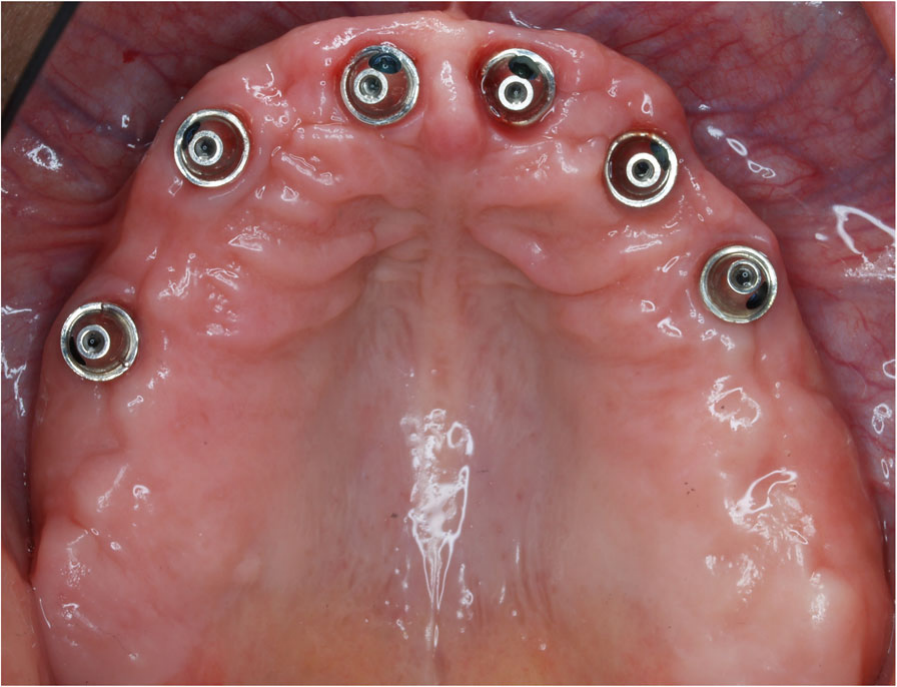
Occlusal view of implant-abutments 3 months post-surgery at the first removal of the temporary restoration

Three months post-surgery, the temporary restorations were removed for the first time (Fig. 4), ISQ and PT values were measured, and the final prosthetic protocol was performed if all implants were osseointegrated.

**Fig. 5:**
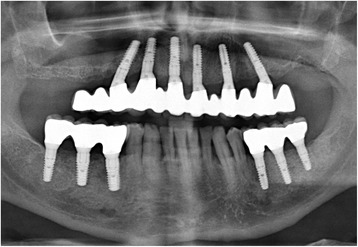
One-year post-surgery panoramic radiograph with final restoration

Changes in marginal bone level were measured using the routinely made digital panoramic radiographs if these were available. The measurement tool was calibrated with the known respective implant length. To evaluate the bone loss, the difference was formed between the bone level at follow-up examination (Fig. 5) and at implant placement which is the baseline.

### Success criteria

An implant was considered as successful if it fulfilled its function without pain or discomfort or clinically detectable mobility and if no peri-implant radiolucency or peri-implant infection was detectable.

### Data analysis

Descriptive statistics, including mean values and standard deviations, were calculated for the continuous parameters using SPSS software (ver. 17.0; SPSS Inc., Munich, Germany).

The measured values were tested for normal distribution with the Kolmogorov-Smirnov goodness-of-fit test. *t* test or nonparametric test was used for the evaluation of differences between dependent or independent samples.

The null hypothesis was that there is a significant difference between measured parameters between tilted and axially inserted implants. The alternative hypothesis was that the differences would be purely random. A significance level of 5% was determined as statistically significant.

To assess the suitability of the two stability parameters ISQ and PT values as potential predictors for the risk of non-osseointegration of immediately loaded splinted maxillary implants in this collective, sensitivity values were plotted against complementary specificity values in receiver operating characteristic (ROC) curves [13, 14]. The area under the curve (AUC) of the ROC analysis is a measure for the quality of the parameter analyzed as a prognostic test. An area of 1 represents a perfect test; an area of 0.5 represents an ineffective test.

## Results

Ten patients with a mean age at implant insertion of 64 ± 11.3 years (range 38 to 81 years; six women, four men) were included. Sixty titanium screw implants (Table 1) were inserted and immediately loaded between 09/2009 and 01/2013.

**Table 1:** Diameters and lengths of immediately loaded implants

	Diameter	Length			
		10 mm	12 mm	14 mm	16 mm
Axial	3.5	3	13	3	–
	4.0	2	7	12	–
Tilted	3.5	–	2	4	–
	4.0	1	4	8	1

Seven patients had remaining teeth until implant surgery (two patients with 4, four patients with 7, and one patient with 12 teeth). Twenty-one (35%) of the 60 immediately loaded implants where inserted in fresh extraction sockets. Six implants in each patient were splinted by the provisional prosthesis on the day of surgery. The opposing dentition was natural teeth (*n* = 4 patients), implant-supported fixed prostheses (*n* = 4 patients), or natural teeth combined with additional implants (*n* = 2 patients). All patients analyzed showed at least opposite dentition with at least the first molar of the mandible replaced on both sides.

### Osseointegration

Three of 60 immediately loaded implants (5%) in three patients were not osseointegrated after first removal of the temporary restorations 3 months after surgery (1 implant among the 40 axial implants [2.5%] and 2 implants among the 20 tilted implants [10%]).

The lost axial implant (12 × 4 mm, ISQ 68, PT value −2) was inserted in a fresh extraction socket in the patient with the most remaining teeth before implant surgery. In this patient, the temporary restoration broke two times.

The two non-osseointegrated tilted implants were both 14 × 4 mm. One was inserted in a maxilla which was edentulous for several years (ISQ 68, PT value −4). The other tilted implant was inserted in a maxilla with seven remaining teeth (ISQ of 49 and a PT value of +1). This implant was located with its apical half in the extraction socket of an immediately extracted canine. All failed implants were immediately replaced with implants of a larger diameter or length. All replaced implants healed load free and transmucosal. In both cases of the two non-osseointegrated tilted implants, the provisional prostheses were shortened but a cantilever extension of one molar width was left since the other implants were osseointegrated at this time. The final prosthesis procedure for the three patients with initial failures started 6 months after the first implant insertion, but the patients were functionally restored with a fixed prosthesis over the entire time.

**Fig. 6:**
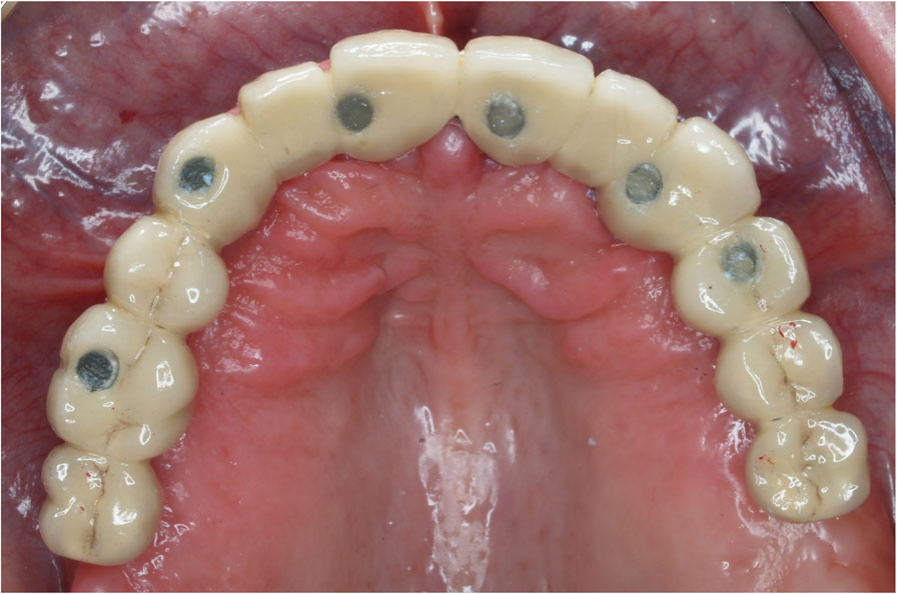
Occlusal view of the final restoration. In this case, with the longest cantilever extension on a final restoration within this collective

**Fig. 7:**
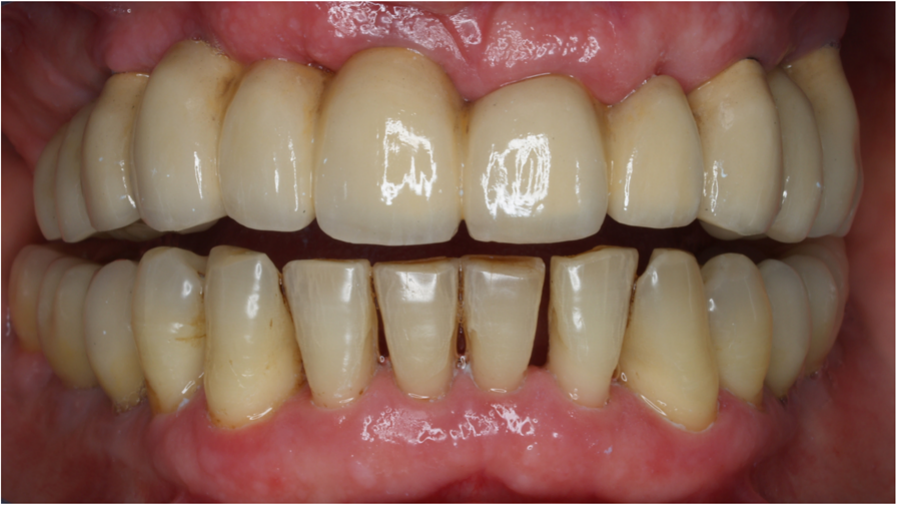
Vestibular view of the final restoration

After the temporary restoration with a fixed prosthesis, all 10 patients selected a fixed final restoration. These consisted of a cast metal framework with a full ceramic veneering including the replacement of at least the second premolars. They were made after a new impression on the abutment level (Figs. 6 and 7). The neck of the 20 tilted distal implants was positioned in region 4 (*n* = 5 implants), region 5 (*n* = 11 implants), and region 6 (*n* = 4 implants). The length of the distal cantilevers had a mean of 7.5 ± 4.1 mm (range 2.0 to 15.5) and replaced a premolar (*n* = 5), a molar (*n* = 5), or two premolars (*n* = 3). Seven times the distal cantilevers did not exceed the tooth under which the distal implant was positioned, leading to very small cantilevers in a range from 2 to 3.5 mm.

The follow-up was 64 ± 13 months (range 42 to 84 months; seven patients ≥5 years, two patients ≥4 years, one patient = 3.5 years) (Table 2).

**Table 2:** Life table of implants

Period	# of implants	# of failures	Survival rate (%)	Cumulative survival rate (%)
0 to 3 months	60	3	95	95
3 to 6 months	57	0	100	95
6 to 9 months	57	0	100	95
9 to 12 month	57	0	100	95
1 year	57	0	100	95
2 years	57	0	100	95
3 years	57	0	100	95
4 years	51	0	100	95
5+ years	40	0	100	95

Except the three failures after the first 3 months, no more failures were recorded and no technical complications occurred at the final restorations. The overall cumulative implant survival rate is 95% (Table 2).

### Implant stability parameters

The mean PT value for osseointegrated implants after 3 months (*n* = 57) was significantly lower (*p* < 0.001), and their ISQ significantly higher (*p* < 0.001) than their means at baseline. Separated into axial (*n* = 39) and tilted (*n* = 18) implants, the differences were also significant (*p* < 0.005) (Tables 3 and 4).

**Table 3:** Mean Periotest values (PT) of survived axial and tilted implants

Collective	PT value		*P*
	At insertion	3 months	
Total (*n* = 57)	−1.8 ± 2.4 Range −8 to 1	−3.5 ± 1.6 Range −7 to −1	<0.001
Axial (*n* = 39)	−2.0 ± 2.5 Range −8 to 1	−3.4 ± 1.5 Range −6 to −1	<0.005
Tilted (*n* = 18)	−1.4 ± 2.1 Range −6 to 1	−3.6 ± 1.7 Range −6 to −1	<0.005

**Table 4:** Mean implant stability quotients (ISQ) of survived axial and tilted implants

Collective	ISQ		*P*
	At insertion	3 months	
Total (*n* = 57)	61.3 ± 7.8 Range 44 to 73	70.8 ± 5.5 Range 56 to 85	<0.001
Axial (*n* = 39)	61.6 ± 7.5 Range 49 to 73	70.7 ± 5.4 Range 56 to 85	<0.001
Tilted (*n* = 18)	60.6 ± 8.7 Range 44 to 72	71.1 ± 5.9 Range 62 to 83	<0.001

Neither the PT value nor the ISQ differed statistically significantly between the axial and tilted implants neither at the baseline examination or after 3 months.

The AUC of the intraoperative-measured PT values was 0.503, with a 95% confidence interval of 0.130–0.876 (*p* = 0.986). The ISQ-AUC was 0.506, with a 95% confidence interval of 0.148–0.864 (*p* = 0.973).

### Marginal bone loss

Bone loss was measured at all 57 osseointegrated implants after 1 year (Table 5) with no statistical significance regarding the implant site (mesial/distal) and the implant inclination (axial/tilted). In 51 implants, an additional bone loss was measured. In contrast to the radiological examination after 1 year, the second radiological examination was not obtained at an identical period. These control radiographs were made at a mean of 55 ± 14 months (range 40 to 84 months; one patient after 7 years, two patients after 5.5 years, one patient after 4.5 years, two patients after 4 years, three patients after 3.5 years) after loading with no statistical significance regarding the implant site and the implant inclination.

**Table 5:** Marginal bone loss measured in mm

Collective	Mesial		Distal	
	1 year	55 months (40–84)	1 year	55 months (40–84)
Total	−0.57 ± 0.46 Range −1.3 to 0.1 (*n* = 57)	−0.81 ± 0.67 Range −2.6 to 0.2 (*n* = 51)	−0.43 ± 0.41 Range −1.6 to 0.1 (*n* = 57)	−0.81 ± 0.74 Range −3.1 to 0.4 (*n* = 51)
Axial	−0.57 ± 0.46 Range −1.3 to 0.3 (*n* = 39)	−0.90 ± 0.68 Range −2.6 to 0.2 (*n* = 35)	−0.41 ± 0.41 Range −1.6 to 0.1 (*n* = 39)	−0.80 ± 0.76 Range −2.6 to 0.4 (*n* = 35)
Tilted	−0.56 ± 0.46 Range −1.3 to 0.1 (*n* = 18)	−0.62 ± 0.64 Range −2.1 to 0.1 (*n* = 16)	−0.51 ± 0.41 Range −1.0 to 0.1 (*n* = 18)	−0.81 ± 0.72 Range −3.1 to 0.0 (*n* = 16)

## Discussion

The overall implant survival rate of 95% is slightly lower than the reported mean survival rates of the concept of tilted implants and immediate loading in edentulous jaws [11] but still close to them and maybe more comparable to investigations in which implants were also immediately loaded in the edentulous maxilla which were partly placed in fresh extraction sites [15, 16]. Nevertheless, what is remarkable is the two lost tilted implants (*n* = 2 of 20). In some reviews, there seems hardly to be a difference in the survival rate between axial and tilted implants [5, 11]. The potentially higher implant loss rate in this study might be due to the limited number of tilted implants.

In 30% of the patients (*n* = 3 of 10), one implant failed. There might be some reasons which could be responsible:

In the present study, one implant failed with a low primary stability. That confirms the assumption that a high primary stability is an important precondition for immediate loading [10]. However, the two other lost implants had high stability parameters. As shown by the low AUC values, the ISQ and PT values were unspecific parameters and unsuitable as a predictor for the risk of non-osseointegration in this collective, and this is in line with other studies [17, 18].

Another failure occurred in a situation where the provisional prostheses broke twice so that this implant might have been overloaded. Two of the failed three implants were completely or partially inserted in fresh extraction sockets, and studies have shown that this is an additional risk for implant failure in immediate loading in edentulous maxillae [16, 19].

That we have not found a significant difference in bone loss between straight and tilted implants is in line with the literature [5, 20]. In both reviews, the differences in bone loss after 12 months are in a range below a tenths of a millimeter and most probably not clinically relevant. It should be taken into account that the level of evidence of most studies is rather low due to the lack of randomized studies and the non-systematic use of a standardized technique to obtain a reproducible bone loss measurement [5, 11, 20]. This is a limit of the present study as well with a single cohort and measurements on digital panoramic radiographs and with irregular time intervals of the second measurement. This could explain that in some cases, even a bone growth was measured (up to 0.4 mm). Another limit of this study is the rather small patient group.

Between baseline and first removal of the temporary restoration after 3 months, the mean ISQ increased and the mean PT value decreased significantly in the axial and tilted implants. This is in contrast to some other studies which evaluated no significant differences of stability parameters between primary and secondary stability with immediate loading in edentulous maxilla [16, 21, 22].

The present study shows that immediate splinted loading on six implants with tilted distal implants is a potential predictable treatment modality for edentulous maxilla even if extraction of remaining teeth and simultaneous implant placement is performed or if very limited bone is available in very atrophic jaws. Immediate implant placement and fractures of provisional prostheses seem to increase the risk of implant failure.

Immediate loading in edentulous maxilla with tilted implants could have a higher risk of initial implant failure, but treatment is less time consuming, less invasive, and in case of immediate implantation may be more comfortable and enhance or restore life quality. If a patient is informed in detail, this protocol seems to have an adequate success probability and treatment of choice for specific situations and patient’s need.

## Conclusions

Within the limits of this small group (*n* = 10 patients/60 implants), the failure rate of the analyzed implant system (*n* = 3 respective 5% implant loss) seems to be comparable with other immediate-loading protocols. On the other side, the implant loss rate of tilted implants (*n* = 2 of 20) in the atrophic upper jaw was quite high, but still, the aimed treatment concept could be achieved in every patient. The analyzed combination of implants, abutments, and materials for the provisional restorations seems to be suitable in the here chosen clinical setting for immediate loading, in part even in combination with immediate implantation.

## Abbreviations

AUC: Area under the curve
ISQ: Implant stability quotient
PT: Periotest
ROC: Receiver operating characteristic
